# Autoimmunity and pulmonary hypertension in patients with Graves’ disease

**DOI:** 10.1007/s00380-014-0518-3

**Published:** 2014-05-18

**Authors:** Tetsuro Sugiura, Shigeo Yamanaka, Hiroaki Takeuchi, Norihito Morimoto, Mikio Kamioka, Yoshihisa Matsumura

**Affiliations:** Department of Laboratory Medicine, Kochi Medical School, Kohasu Oko-cho, Nankoku City, Kochi 783-8505 Japan

**Keywords:** Pulmonary hypertension, Hyperthyroidism, Echocardiography, Autoimmunity

## Abstract

A link between hyperthyroidism and pulmonary hypertension has been reported, but the underlying mechanisms of these two conditions have not been clearly identified. The aim of this study was to determine the clinical correlates of pulmonary hypertension in patients with Graves’ disease. Among 50 consecutive patients with Graves’ disease referred for echocardiography, 18 patients (36 %) had pulmonary hypertension measured by continuous-wave Doppler echocardiography (pulmonary artery systolic pressure >35 mmHg). The patients with pulmonary hypertension had significantly higher pulmonary vascular resistance (PVR), cardiac output and thyroid-stimulating hormone receptor antibody (TRAb) compared to those without (*p* < 0.001, *p* = 0.028 and *p* < 0.001, respectively). Pulmonary artery systolic pressure had a good correlation with TRAb (*r* = 0.74, *p* < 0.001), but was not related to free T4 (*r* = 0.12, *p* = 0.419) and free T3 (*r* = 0.22, *p* = 0.126). To determine the important variables present in patients with Graves’ disease that may be related to pulmonary artery systolic pressure, 4 variables (PVR, cardiac output, TRAb and free T3) were used in the multivariate analysis. In addition to PVR (standard regression coefficient = 0.831, *p* < 0.001) and cardiac output (standard regression coefficient = 0.592, *p* < 0.001), TRAb (standard regression coefficient = 0.178, *p* < 0.001) emerged as a significant variable related to pulmonary artery systolic pressure. Thus, in addition to the effect of thyroid hormone on the cardiovascular system, autoimmune-mediated pulmonary vascular remodeling may play a role in Graves’ disease-linked elevated pulmonary artery systolic pressure.

## Introduction

Characteristic clinical manifestations of hyperthyroidism are those resulting from the effects of thyroid hormone on the cardiovascular system [1, 2]. Common cardiovascular signs of hyperthyroidism include sinus tachycardia, atrial arrhythmias, increased cardiac output, widened pulse pressure and elevated pulmonary artery pressure [1–7]. Although Doppler echocardiography may be imprecise in determining actual pressures compared to invasive evaluation, it is performed as a noninvasive screening test that can detect pulmonary hypertension [8–11]. A high prevalence of pulmonary hypertension, detected by Doppler echocardiography, has been observed in patients with hyperthyroidism [4–6]. However, pathophysiologic link between hyperthyroidism and pulmonary hypertension remains unclear. Because studies with a limited number of patients have looked into the association between hyperthyroidism and elevated pulmonary artery pressure, we designed a study to determine the clinical correlates of elevated pulmonary artery systolic pressure in patients with Graves’ disease.

## Subjects and methods

### Patients’ characteristics

This observational study was performed in 59 consecutive patients with recently diagnosed Graves’ disease (within 12 weeks of diagnosis) recruited from the echocardiography laboratory of the Kochi Medical School Hospital. All the patients were referred to the echocardiography laboratory by physicians from the endocrinology outpatient clinic. The main inclusion criteria for enrolment were no history of heart disease (congenital heart disease, coronary artery disease, valvular heart disease or cardiomyopathy), lung disease, liver disease, or collagen vascular disease. We excluded patients who were taking vasoactive drugs (calcium channel blockers, alpha-adrenergic blockers, angiotensin converting enzyme inhibitors and angiotensin receptor blockers). Lung disease was excluded by chest radiography and careful chart reviews. Patient evaluation included a medical history, physical examination and laboratory studies. The diagnosis of Graves’ disease was made by the findings of sustained hyperthyroidism; serum-free thyroxine (T4) level >1.79 ng/dL, free triiodothyronine (T3) >4.0 pg/mL with concomitant decrease in thyroid-stimulating hormone (TSH) levels, elevated TSH receptor antibody (TRAb) levels (>2 IU/L ), increased blood flow in the thyroid gland on ultrasonography and a diffuse goiter. All samples were prepared and analyzed in accordance with the ethical recommendations of the hospital’s committee on human research and written informed consent was obtained from all subjects.

### Measurements

Blood samples were collected from the antecubital vein within 24 h of the echocardiography. Serum albumin was measured by a dye-binding bromocresol green procedure and C-reactive protein (CRP) was measured by latex agglutination immunoassay using a JCA-BM2250 analyzer (Japan Electron Optics Laboratory, Tokyo, Japan). White blood cells were measured by the direct current detection method using a Sysmex SE 9000 (Sysmex, Kobe, Japan). Blood samples were also analyzed for free T4, free T3, TSH and TRAb by the electrochemiluminescence immunoassay using Modular Analytica E170 (Roche Diagnostics, Rotkreuz, Switzerland).

### Echocardiography

Transthoracic echocardiography was performed with a Philips ultrasound iE 33 phased-array sector scanner (Philips, Bothell, USA) or a Sequoia 512 (Siemens, Erlangen, Germany) using a 3.75 or 2.5 MHz transducer by an experienced echocardiographer. Conventional M-mode and 2D echocardiographic measurements were performed in the standard manner [12–14]. Doppler recordings of tricuspid regurgitation were performed in the apical 4-chamber view. The regurgitation jet was recorded by means of continuous-wave Doppler, and the maximum velocity was used to calculate the transtricuspid pressure gradient by means of the modified Bernoulli equation [13]. Pulmonary artery systolic pressure was calculated as the sum of the transtricuspid pressure gradient in conjunction with an echocardiographic estimation of right atrial pressure. Echocardiographic estimation of right atrial pressure was performed based on inferior vena cava size and collapsibility, according to the previously established criteria [15]. Measurement of cardiac output was performed at rest from the apical 2D echocardiograms using a modified Simpson technique and multiplying stroke volume by heart rate. Pulmonary vascular resistance (PVR) was estimated by Doppler echocardiography using the equation proposed by Lindqvist et al.: PVR = (pulmonary artery mean pressure − 10)/cardiac output, where pulmonary artery mean pressure = pulmonary artery systolic pressure × 0.61 + 2 mmHg [16, 17]. All classic views were recorded on videotape for subsequent analysis by physicians who were board certified in echocardiography.

### Statistical analysis

Results are reported as the mean value ± standard deviation. Statistical analysis between the 2 groups was performed by the Student’s *t* test or the Mann–Whitney test for continuous variables and the Fisher exact probability test for discrete variables. Correlation coefficients between the 2 continuous variables were obtained using the linear regression analysis. Multiple regression analysis was performed to identify the variables independently related to pulmonary artery systolic pressure. A *p* value <0.05 was considered significant.

## Results

**Fig. 1:**
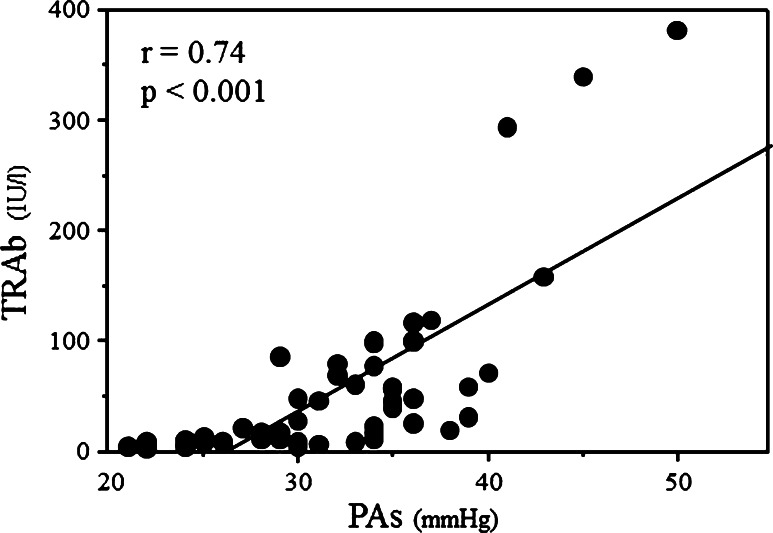
Correlation between pulmonary artery systolic pressure and TRAb. *PAs* pulmonary artery systolic pressure, *TRAb* thyroid-stimulating hormone receptor antibody

Among 59 patients with Graves’ disease, pulmonary artery systolic pressure could not be determined in 9 patients (18 %) due to the absence of tricuspid regurgitation. Therefore, this study consists of 50 patients. Eighteen patients (36 %) had pulmonary hypertension (pulmonary artery systolic pressure >35 mmHg). There were no significant differences in age, gender distribution, cardiac output, free T4, free T3, TSH, creatine phosphokinase, CRP levels, cholesterol, serum albumin, white blood cells or incidence of medical therapy (beta-blocker and/or thiamazole) between patients with and without pulmonary hypertension (Tables 1, 2). Cardiac output, PVR and TRAb were significantly higher in patients with pulmonary hypertension compared to those without. Pulmonary artery systolic pressure had a good correlation with TRAb (*r* = 0.74, *p* < 0.001) (Fig. 1), but was not related to free T4 (*r* = 0.12, *p* = 0.419) and free T3 (*r* = 0.22, *p* = 0.126).

**Table 1:** Clinical characteristics

	Pulmonary hypertension		*p* value
	Present (*n* = 18)	Absent (*n* = 32)	
Age (years)	54 ± 12	49 ± 14	0.135
Men/women	4/14	6/26	0.768
Cardiac output (l/min)	6.33 ± 1.37	5.55 ± 1.48	0.028
PVR (WU)	2.52 ± 0.57	1.79 ± 0.55	<0.001
Medication			
Beta-blocker	11 (61 %)	19 (59 %)	0.904
Thiamazole	14 (78 %)	24 (75 %)	0.825

*PVR* pulmonary vascular resistance

**Table 2:** Biochemical and serologic findings

	Pulmonary hypertension		*p* value
	Present (*n* = 18)	Absent (*n* = 32)	
Free T4 (ng/dL)	3.61 ± 1.69	3.40 ± 1.91	0.492
Free T3 (pg/mL)	11.33 ± 5.32	9.21 ± 5.52	0.125
TSH (µU/mL)	0.008 ± 0.005	0.010 ± 0.013	0.679
TRAb (lU/L)	115 ± 110	29 ± 31	<0.001
Creatine phosphokinase (U/L)	65 ± 52	52 ± 33	0.419
C-reactive protein (mg/dL)	0.128 ± 0.149	0.156 ± 0.152	0.332
Cholesterol (mg/dL)	163 ± 43	158 ± 34	0.694
Albumin (g/L)	41.4 ± 4.5	40.0 ± 5.0	0.266
White blood cells (/µL)	5811 ± 1655	5381 ± 1597	0.374

*T3* triiodothyronine, *T4* thyroxine, *TRAb* thyroid-stimulating hormone receptor antibody, *TSH* thyroid-stimulating hormone

To determine the important variables present in patients with Graves’ disease that may be related to pulmonary artery systolic pressure, 4 variables (PVR, cardiac output, TRAb and free T3) were used in the multivariate analysis. From the analysis, in addition to PVR (standard regression coefficient = 0.831, *p* < 0.001) and cardiac output (standard regression coefficient = 0.592, *p* < 0. 001), TRAb (standard regression coefficient = 0.178, *p* < 0.001) emerged as a significant variable related to pulmonary artery systolic pressure.

## Discussion

The effects of thyroid hormone on the heart and vasculature are increase in heart rate, left ventricular contractility and blood volume, and decrease in systemic vascular resistance [1, 4]. However, the symptoms and signs of Graves’ disease result not only from direct and indirect effects of hyperthyroidism but also caused by autoimmune process of Graves’ disease [1–6]. Association between hyperthyroidism and pulmonary hypertension has been described, but the underlying mechanisms of these 2 conditions have not been clearly identified. Among our patients with Graves’ disease, elevated pulmonary artery systolic pressure was found in 36 % by Doppler echocardiography, a widely used method for estimating pulmonary artery systolic pressure, which is consistent with previous reports that elevated pulmonary artery systolic pressure is a relatively common complication in patients with Graves’ disease.

Increase in cardiac output and/or elevated PVR are the 2 major pathophysiologic factors determining the elevation of pulmonary artery systolic pressure. The factors related to the increased cardiac output in patients with hyperthyroidism are increases of heart rate and myocardial contractility due to the effects of hyperthyroidisms on the sympathetic nerve system, and a decrease of the systemic vascular resistance largely due to excessive nitric oxide production [18]. Furthermore, increase in cardiac output is also observed in hyperthyroidism due to an increase in blood volume resulting from increased net tubular reabsorption of sodium [19]. In contrast to the effect of thyroid hormone to decrease systemic vascular resistance, it has been suggested that pulmonary vascular resistance is not decreased by hyperthyroidism [20]. Thyroid hormones may affect the pulmonary vasculature by affecting the sympathetic nervous system or alteration of the energy metabolism; enhanced catecholamine sensitivity, decrease in cholinergic tone, increased metabolism of the intrinsic pulmonary vasodilating substances and decreased metabolism of the vasoconstrictors, which together will account for an increase in the PVR [1–3, 21, 22]. Moreover, an increase in cardiac output could cause endothelial damage, and hence increase PVR. In this study, the patients with pulmonary arterial hypertension had significantly higher cardiac output and PVR compared to those without. Our data indicate that increased cardiac output and PVR due to excessive thyroid hormone were the factors associated with an elevation of pulmonary artery pressure in our patients with Graves’ disease.

Antithyroid antibodies are a marker for generalized immune activation. Chu et al. [23] demonstrated a high prevalence of autoimmune thyroid disease in prospective series of pulmonary hypertension and suggested an autoimmune pathogenetic link between these 2 conditions. On the other hand, Merces’ reported that elevated cardiac output partially explains the pathogenesis for pulmonary hypertension in hyperthyroidism, and they did not support the autoimmune pathogenesis for pulmonary hypertension [4]. In our patients with Graves’ disease, despite no significant correlation between pulmonary artery systolic pressure and free T3, TRAb had a good positive correlation with pulmonary artery systolic pressure. Graves’ disease is an autoimmune disease in which excessive amount of thyroid hormone is produced by TRAb. TRAb binds to the TSH receptors and chronically stimulates them resulting in an abnormally high production of thyroid hormones. Therefore, almost all patients with Graves’ hyperthyroidism have detectable TRAb that has a major pathogenic role in Graves’ disease; persistent elevation correlates with disease activity, while remission is usually accompanied by a decrease in their activity [24, 25]. The discrepancy between the 2 studies is probably due to the lower incidence of autoimmune hyperthyroidism in Merces’ study (54 % for antiperoxidase antibody and 35 % for antithyroglobulin antibody) compared to our study (100 % for TRAb). Nicolls et al. [26] reported the role of autoimmunity in the development of pulmonary hypertension. They indicated the endothelial cell destruction by immune-mediated injury, which results in the generation of rapidly proliferative apoptosis-resistant endothelial cells, leading to vascular remodeling and hence the development of pulmonary hypertension. Considering the results that TRAb was found to be an important factor associated with elevated pulmonary artery systolic pressure by the multivariate analysis, our data indicate that pathogenic autoantibodies targeting endothelial cells could cause endothelial damage or dysfunction and play a role in Graves’ disease-linked elevated pulmonary artery systolic pressure.

Three limitations of our study should be addressed. First, pulmonary artery systolic pressure was estimated non-invasively. Although echocardiography may be imprecise in determining actual pressure compared to invasive evaluation of pulmonary artery systolic pressure, considering the fact that echocardiography may underestimate pulmonary artery systolic pressure in patients with severe pulmonary hypertension and overestimate pulmonary artery systolic pressure in patients with normal pressures, our conclusion would probably not be altered by an invasive measurement. Moreover, invasive measurement is not ethical or practical in outpatients. Second, we may have underestimated the prevalence of pulmonary hypertension because pulmonary artery systolic pressure could not be determined in 9 patients (18 %) due to the absence of tricuspid regurgitation. Nonetheless, data obtained by cardiac catheterization show that moderate or severe pulmonary hypertension is almost always associated with tricuspid regurgitation. Third, medical therapy was started in most of the patients at the time of cardiovascular evaluation because clinician judged it was necessary to start treatment. However, all the patients were in hyperthyroid state with elevated TRAb and remission is usually accompanied by decrease in TRAb activity.

In conclusion, in addition to the effect of thyroid hormone on the cardiovascular system, autoimmune-mediated pulmonary vascular remodeling may play a role in Graves’ disease-linked elevated pulmonary artery systolic pressure.
